# Supplementary material for: FERN – a Java framework for stochastic simulation and evaluation of reaction networks
Source: BMC Bioinformatics. 2008 Aug 29;9:356. doi: 10.1186/1471-2105-9-356 (PMC2553347; doi:10.1186/1471-2105-9-356)
Supplement: Additional file 1 — FERN distribution, Version 1.3. This archive contains the FERN source code and binaries as well as documentation and example models in FernML and SBML. [file 1471-2105-9-356-S1.zip › fern/doc/javadoc/index-files/index-11.html]

L-Index


---


|  |  |  |
| --- | --- | --- |
| **Overview** \| Package \| Class \| Use \| **Tree** \| **Deprecated** \| **Index** \| **Help** | |  |
| **PREV LETTER**   **NEXT LETTER** | **FRAMES**    **NO FRAMES**     **All Classes** |  |


A B C D E F G H I J L M N O P R S T U V 

---


## **L**

**LacYComplete** - Class in fern.example: The LacZ/LacY model of procaryotic gene expression proposed by [1] is simulated. **LacYComplete()** - Constructor for class fern.example.LacYComplete: **LacYHistogramDistances** - Class in fern.example: The LacZ/LacY model of procaryotic gene expression proposed by [1] is simulated. **LacYHistogramDistances()** - Constructor for class fern.example.LacYHistogramDistances: **LacZ** - Class in fern.example: The LacZ/LacY model of procaryotic gene expression proposed by [1] is simulated. **LacZ()** - Constructor for class fern.example.LacZ: **LeapObserver** - Class in fern.simulation.observer: Observes the amount of given molecule species at each `step`. **LeapObserver(Simulator, String...)** - Constructor for class fern.simulation.observer.LeapObserver: Creates the observer with a given simulator and given species **loadHistogram(File)** - Static method in class fern.tools.NumberTools: Reads a previously saved histogram from a file. **loadLabelsAndLineStyles(String[], String[])** - Method in class fern.tools.gnuplot.Axes: Adds new labels and styles to the axes object. **loadNetwork(Network)** - Method in class fern.cellDesigner.ui.MainFrame: **loadNetwork(Network)** - Method in class fern.cytoscape.ui.MainFrame: **loadNetwork(File)** - Static method in class fern.tools.NetworkTools: Loads a network from file identifying the type (FernML/SBML).

---


|  |  |  |
| --- | --- | --- |
| **Overview** \| Package \| Class \| Use \| **Tree** \| **Deprecated** \| **Index** \| **Help** | |  |
| **PREV LETTER**   **NEXT LETTER** | **FRAMES**    **NO FRAMES**     **All Classes** |  |


A B C D E F G H I J L M N O P R S T U V 

---
